# Successful Eradication of Feline Coronavirus in Breeding Catteries Paves the Way to Prevent Feline Infectious Peritonitis †

**DOI:** 10.3390/v18060614

**Published:** 2026-05-28

**Authors:** Luna Vanden Buijs, Jolien Van Cleemput, Emmanuel Abatih, Hans Nauwynck

**Affiliations:** 1 Department of Translational Physiology, Infectiology and Public Health, Faculty of Veterinary Medicine, Ghent University, Salisburylaan 133, 9820 Merelbeke-Melle, Belgium; luna.vandenbuijs@ugent.be (L.V.B.); jolien.vancleemput@ugent.be (J.V.C.); 2 Department of Mathematics, Computer Science and Statistics, Faculty of Sciences, Ghent University, Krijgslaan 299, 9000 Ghent, Belgium

**Keywords:** feline coronavirus, cattery management, viral shedding, eradication protocol, feline infectious peritonitis

## Abstract

Feline coronavirus (FCoV) is widespread in multi-cat environments. Although most infections are asymptomatic, FCoV may mutate into a virulent form responsible for feline infectious peritonitis (FIP), a progressive and mostly fatal systemic disease. Controlling FCoV circulation in multi-cat environments is challenging, but desirable for feline health and breeding management. In this study, we evaluated the impact of stepwise management protocols with increasing stringency on viral shedding in 20 catteries across Flanders, Belgium. Each cattery was allocated to one of three eradication protocols: voluntary (*n* = 10), stimulatory (*n* = 5) or obligatory (*n* = 5). Rectal swabs were collected at the start (T0) and twice after implementation (T6m = 6 months, T12m = 12 months) and tested by RT-qPCR. With the voluntary protocol, the proportion of shedders decreased significantly from 78.6% at T0 to 62.4% at T6m and 45.5% at T12m, though all catteries remained positive (minimum one shedder). The stimulatory protocol reduced shedding from 70.5% (T0) to 30.5% at T6m and 12.9% at T12m, with two of five catteries achieving a negative status. The obligatory eradication protocol achieved the most pronounced effect, with the shedding declining from 55.5% at the start to 17.1% at T6m and 2.5% at T12m, and four of five catteries testing negative by T12m. These findings demonstrate that cattery-level FCoV control is feasible, with an effectiveness depending on the stringency of management. At the end, FCoV-negative catteries can sell certified FCoV-negative kittens.

## 1. Introduction

Feline coronavirus (FCoV) is an enveloped, positive-sense RNA virus belonging to the family *Coronaviridae* and is ubiquitous among domestic cats worldwide [1,2]. Prevalence studies indicate that around 12.5–100% of the domestic cat population actively sheds the virus in multi-cat environments such as shelters and breeding catteries [3,4,5,6,7,8,9,10,11,12,13,14,15,16,17,18,19]. The virus exists in two pathotypes: less-virulent FCoV and feline infectious peritonitis (FIP)-associated FCoV [20].

The less-virulent FCoV replication occurs initially in enterocytes, often resulting in subclinical infections or mild enteric problems [21,22]. However, in up to 12% of infected cats, mutations within the viral genome lead to altered cell tropism (macrophages), resulting in feline infectious peritonitis (FIP), a progressive and almost invariably fatal disease [23,24,25,26]. Notably, due to the recent availability and high efficacy of antiviral therapies, FIP is nowadays considered a largely treatable disease.

Several risk factors have been identified in association with FCoV infection, including housing density, litter box conditions, and general hygiene practices. Cats living alone in single-cat households show the lowest risk of being FCoV seropositive, whereas those kept in multi-cat environments are significantly more likely to become infected [26]. The type of cat litter material may also influence viral transmission: cat litters based on Fuller’s earth have been shown to inhibit FCoV infection in cell culture experiments, but they failed to completely prevent virus transmission under real-life conditions [4]. Ultimately, one of the most important factors contributing to FCoV spread is the housing of multiple cats sharing litter trays and feeding bowls, which greatly facilitates fecal–oral transmission of the virus [27]. Therefore, maintaining strict hygiene by frequently cleaning litter boxes and providing an adequate number of boxes are important measures to lower the risk of viral transmission.

Managing FCoV infection in multi-cat environments is therefore not only resulting in a reduction in enteric problems but, more importantly, in the prevention of the emergence of FIP. In this study, we describe the implementation and outcome of structured FCoV elimination protocols in multiple breeding catteries. By combining targeted hygiene measures, population management, and virological monitoring, we aimed to achieve and maintain an FCoV-free status. In this context, our approach provides a feasible alternative to antiviral elimination strategies, such as the one described by Addie et al. They administered the antiviral compound GS-441524 for 4 to 7 days to halt fecal FCoV shedding and, as a result, prevent FIP development [28]. However, relying solely on antiviral treatments to suppress viral shedding poses a significant long-term risk, particularly regarding the potential selection and emergence of antiviral-resistant FCoV variants. Implementing structured, preventive management and biosecurity protocols, as evaluated in the present study, offers a more sustainable and robust alternative. This approach effectively interrupts virus circulation and achieves long-term eradication without exerting selective pressure on the viral genome, thereby safeguarding the future efficacy of essential antiviral therapies.

This study aimed to evaluate the effectiveness of stepwise management protocols on FCoV shedding in breeding catteries. Our findings prove that FCoV eradication is feasible by following strict measures. Multi-cat households pose a tough challenge in this context due to the large number of viral shedders, reinfection dynamics, shared litter facilities, and the difficulty of maintaining well-defined biosecurity protocols.

## 2. Materials and Methods

### 2.1. Cats and Catteries

A total of 20 catteries in Flanders (Belgium) participated in this study, with a total of 259 cats that were sampled. Due to the dynamic nature of cattery populations, the number of cats present at any single timepoint fluctuated, with 168 cats sampled at the start (T0).

Within the voluntary protocol group (*n* = 10 catteries), a total of 98 cats were present at the beginning of the study, with a mean baseline size of 9.8 cats per cattery. In the stimulatory protocol group (*n* = 5 catteries), 34 cats were sampled at the start, with a mean baseline cattery size of 6.8 cats. In the obligatory protocol group (*n* = 5 catteries), 36 cats were sampled at the start, with a mean baseline cattery size of 7.2 cats.

Breeders were recruited via phone, e-mail, or personal contact, and informed consent was obtained prior to participation. Catteries were included when they had at least 1 female intact cat and housed at least 3 cats in total. Rectal swabs were collected from all cats older than eight weeks that were housed in each breeding cattery. An approval for this field experiment was given by the Ethics Committee on Animal Research and Testing in the Faculty of Veterinary Sciences at UGent (number 2025-91).

Of the 259 sampled cats, 175 were female (30 neutered, 145 sexually intact) and 84 were male (16 neutered, 68 sexually intact). The age of the cats ranged from 8 weeks to 15 years. All cats were purebred, representing the following breeds: British Shorthair (BSH), Neva Masquerade (NEM), Siberian (SIB), Thai (THA), Cornish Rex (CRX), Bengal (BEN), Russian Blue (RUS), Exotic (EXO), Himalayan (HIM), and Persian (PER).

### 2.2. Management Protocols

Three different management protocols (provided as Supplementary Materials), with increasing levels of stringency, were implemented in participating catteries. All protocols comprised the same core set of measures, but the intensity and rigor with which these measures were implemented varied by protocol. These measures included grouping of cats based on FCoV shedding status, adjustments in screening frequency, reinforcement of general hygiene (including litter box hygiene), the use of bentonite-based cat litter, the identification and isolation of persistent shedders and the screening of potential breeding partners. Each cattery was allocated to one of the three groups according to its willingness and feasibility to comply with the measures.

1. Voluntary FCoV eradication protocol (*n* = 10 catteries): A set of recommended hygiene and management measures, applied on a voluntary basis. The primary objective was to reduce the overall number of FCoV shedders within the cattery population.

2. Stimulatory FCoV eradication protocol (*n* = 5 catteries): A stimulatory protocol including hygiene and management measures that were rigorously enforced. The objective was to achieve and maintain a cattery population in which fewer than 20% of cats were FCoV shedders.

3. Obligatory FCoV eradication protocol (*n* = 5 catteries): An intensified and mandatory protocol, comprising the strict protocol measures with additional restrictions. The objective was to house only non-shedding cats and fully eliminate FCoV from the cattery.

### 2.3. Samples

At least 3 rectal swabs were collected from each cat: one prior to the implementation of the control measures (T0), and two at the following timepoints: T6m (=after 6 months) and T12m (=after 12 months). When necessary (birth litter, identification of a persistent shedder or (re)entry cat), an additional swab was taken in between the timepoints. The 6-month intervals (T0, T6m, T12m) were selected based on the prolonged nature of FCoV shedding and the time required for biosecurity protocols to interrupt virus circulation. The 12-month endpoint confirmed the sustainability of the eradication protocols across a full annual breeding cycle.

Swabs were stored at 4 °C for no longer than 24 h before further processing. Each swab was suspended in 1.5 mL of Dulbecco’s phosphate-buffered saline (dPBS), vortexed thoroughly, and immediately centrifuged at 13,000 rpm for 10 min at 4 °C. A 200 µL aliquot of the resulting supernatant was collected for downstream analysis.

Serological evaluation was not performed in this study because antibody titers indicate past exposure and cannot differentiate active virus shedders from recovered cats.

### 2.4. ORF 1b 5′ RT-qPCR

Viral RNA was extracted from rectal swabs using the IndiSpin Pathogen Kit (Indical Bioscience, Leipzig, Germany) according to the manufacturer’s instructions. DNase I was used to remove DNA contaminants. Dulbecco’s PBS (dPBS) was included as a negative extraction control, with one control per extraction batch. Viral genomic load was assessed using an in-house 5′ RT-qPCR [29] targeting a 137 bp fragment of ORF1b. Amplification was performed with the forward primer ORF1bFW (5′–TGGACCATGAGCAAGTCTGTT–3′) and reverse primer ORF1bRV (5′–CAGATCCATCATTGTGTACTTTGTAAGA–3′) as previously described [29]. This assay was selected because it targets a highly conserved region within the ORF1b gene, ensuring pan-reactive detection of diverse FCoV isolates. Prior to the study, the analytical inclusivity of the assay was rigorously evaluated and validated in our laboratory against a panel of different FCoV strains, including both serotypes (type I and type II) as well as both biotypes (less-virulent FCoV and FIP-associated FCoV). The assay successfully amplified all tested strains, confirming its high diagnostic robustness across heterogeneous viral populations.

Each 20 µL reaction consisted of 10 µL SYBR Green/ROX One-Step RT-qPCR Master Mix (Precision, PrimerDesign, Hampshire, UK), 0.4 µL forward primer, 0.8 µL reverse primer, 5.8 µL RNase/DNase-free water, and 3 µL RNA template. All samples were run in duplicate. Amplification was performed on a QuantStudio 3 Real-Time PCR System (Applied Biosystems, Thermo Fisher Scientific, Waltham, MA, USA) with the following cycling conditions: reverse transcription at 55 °C for 10 min, enzyme activation at 95 °C for 8 min, followed by 40 cycles of denaturation at 95 °C for 10 s and annealing/extension at 58 °C for 60 s. A melting curve analysis (95 °C for 15 s, 60 °C for 60 s, and 95 °C) was included to confirm the specificity of amplification. While dye-based platforms like SYBR Green are sometimes considered less specific than probe-based technologies, this approach was highly robust due to the systematic inclusion of a melting curve analysis for every sample. This quality control step allowed for the definitive verification of the specific amplicon melting temperature, completely mitigating the risk of non-specific background noise or primer–dimer amplification up to the positivity cut-off of cycle threshold (Ct) 35.

Samples with a Ct-value below 35 were considered positive. Each run included three quality controls: a positive control (FIP-associated FCoV I strain Black RNA), a negative control (UP water) and a negative RNA-extraction control (dPBS).

### 2.5. Definitions

Cats were classified as FCoV-positive at a timepoint when viral RNA was detected by RT-qPCR with a Ct-value < 35 in at least one of two duplicates. Cats were defined as persistent shedders when they remained RT-qPCR positive with an interval of 4–6 months in isolation.

Catteries were considered positive at a given timepoint if at least one cat housed in the facility tested positive by RT-qPCR.

### 2.6. Statistical Analysis

All statistical analyses were performed using IBM SPSS Statistics for macOS, Version 30.0 (IBM Corp., Armonk, NY, USA). To account for the repeated-measures design, a linear mixed-effects model (LMM) was used to assess the effect of time on the outcome variable. Time (with three levels: T0, T6m and T12m) was included as a fixed effect, and random intercepts were specified for each cattery to account for within-subject variability. Model assumptions were checked visually. Normality of residuals was inspected via histograms. Linearity and homoscedasticity were checked using residuals vs. predicted values scatterplots. Outliers were examined using boxplots. No violations of model assumptions were detected. Statistical significance was set at *p* < 0.05.

A generalized linear model (GLM) with a binomial distribution and logit link function was used to examine the effect of time on the probability of a successful outcome. The model included the number of successful outcomes (outcome) out of a total number of trials (total) as the dependent variable. Time was entered as a continuous predictor. An offset term (log of total) was included to account for varying numbers of trials. To account for the repeated-measures structure of the data (i.e., multiple observations per participant), Generalized Estimating Equations (GEEs) with subject ID as the clustering variable were used. An independent working correlation structure was specified, and robust (sandwich) standard errors were used. The model was fitted using the Fisher scoring algorithm with a convergence criterion of 1 × 10^−6^. Model fit was evaluated using the Quasi-likelihood under the Independence model Criterion (QIC) and the corrected QIC (QICC). Statistical significance was evaluated using a two-tailed criterion of *p* < 0.05.

To identify potential variables associated with the dynamics of FCoV shedding, a comprehensive risk factor analysis was performed using a linear mixed-effects model. This risk factor analysis evaluated both cattery-level factors, specifically the number of cats within the cattery, baseline infection prevalence (T0), and detected viral loads, as well as individual cat-level factors, including age, sex, and breed. To assess the robust efficacy of each management approach, the risk factor analysis was stratified per protocol group. In each multivariable model, the individual cat was treated as a repeated measure across the three study timepoints. A random effect for cattery identification was incorporated into the models to control for environmental clustering within facilities. Statistical significance was set at *p* < 0.05.

## 3. Results

The effect of each protocol was assessed by comparing the proportion of FCoV shedders before (T0) and after implementation (T6m and T12m). The individual management (in/out/isolation/birth of kittens) and virological results of the different cats in each cattery over time can be found in Supplementary Figures S1–S3.

### 3.1. Positive Impact of Voluntary FCoV Eradication Protocol

**Figure 1:**
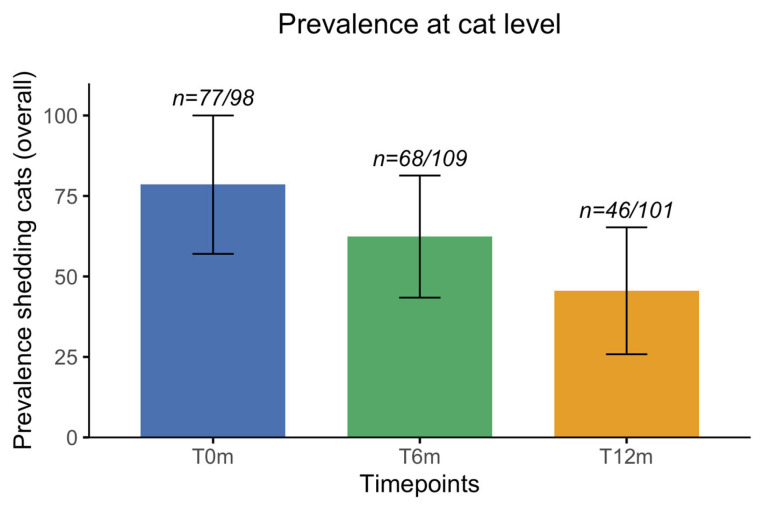
Effect of a voluntary FCoV eradication program on the FCoV shedding in catteries. Bars represent the percentage of cats shedding FCoV at the three sampling timepoints (T0, T6m, T12m). Numbers above the bars represent the ratios of positives to the total tested. Error bars represent the weighted standard deviation (SD), reflecting the variation in prevalence between catteries. The overall effect of time was statistically significant (GLM with binomial distribution and logit link, *p* < 0.001).

In Figure 1, it is shown that at the start (T0), 78.6% of the cats (*n* = 77/98) were shedding FCoV. After implementation of the voluntary protocol, the proportion of shedders decreased to 62.4% (*n* = 68/109) at T6m and 45.5% (*n* = 46/101) at T12m. The overall reduction across timepoints was statistically significant (GLM, *p* < 0.001).

A linear mixed-effects model confirmed a significant negative effect of time on the outcome (B = −16.147, SE = 6.034, *p* = 0.015). Residual diagnostics indicated that model assumptions were met and no major outliers were present. Similarly, a generalized linear model (GLM) with a binomial distribution and logit link function revealed a significant effect of time on the probability of shedding (B = −0.735, SE = 0.1077, *p* < 0.001). The odds of shedding decreased over time, with an odds ratio of 0.480 (95% CI [0.388, 0.592]).

Despite significant reductions in the number of shedders per cattery, the prevalence at the cattery level remained unchanged throughout the study: all 10 catteries had at least 1 FCoV-shedding cat at every timepoint (Table 1). Nevertheless, nine out of ten catteries achieved their objective of reducing the overall number of shedders.

The success of the voluntary protocol was significantly associated with specific variables. A higher baseline infection prevalence within the cattery was significantly correlated with FCoV shedding (*p* = 0.016). Furthermore, the cat’s breed was identified as a highly significant risk factor influencing the shedding outcomes over time (*p* < 0.001). The number of cats, viral loads, age, and sex remained non-significant.

### 3.2. Positive Impact of Stimulatory FCoV Eradication Protocol

**Figure 2:**
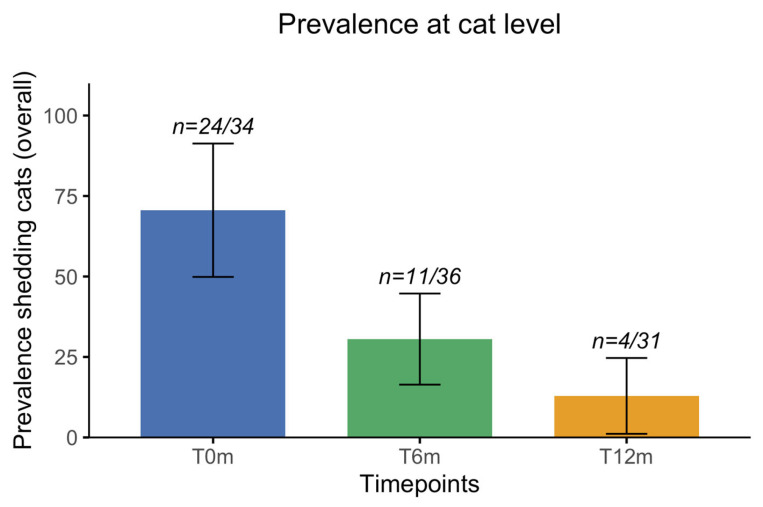
Effect of a stimulatory FCoV eradication program on the FCoV shedding in catteries. Bars represent the percentage of cats shedding FCoV at the three sampling timepoints (T0, T6m, T12m). Numbers above the bars represent the ratios of positives to the total tested. Error bars represent the weighted standard deviation (SD), reflecting the variation in prevalence between catteries. The overall effect of time was statistically significant (GLM with binomial distribution and logit link, *p* < 0.001).

At the start of the study (T0), 70.5% of cats (*n* = 24/34) were shedding FCoV (Figure 2). Following implementation of the protocol, the proportion decreased to 30.5% (*n* = 11/36) at timepoint T6m and 12.9% (*n* = 4/31) at timepoint T12m. The overall reduction across the timepoints was statistically significant (GLM, *p* < 0.001).

A linear mixed-effects model confirmed a significant effect of time on the outcome (B = −27.55, SE = 5.81, *p* = 0.001). Residual diagnostics indicated that model assumptions were met and no major outliers were detected. The generalized linear model (binomial, logit link) also showed a significant decrease in the probability of shedding over time (B = −1.45, SE = 0.29, *p* < 0.001) with an odds ratio of 0.24 (95% CI [0.14, 0.41]).

At the cattery level, the prevalence remained 100% at T0 and T6m but decreased to 60% at T12m (Table 2). Three out of five catteries achieved their objective to house less than 20% of shedding cats. In fact, two out of five catteries were even completely FCoV-free after 12 months of implementation.

The multivariate analysis demonstrated that none of the evaluated risk factors had a significant association with FCoV shedding over time (*p* > 0.05). The number of cats, baseline infection prevalence, viral loads, age, sex, and breed did not significantly influence the successful eradication of the virus under this strict protocol.

### 3.3. Positive Impact of Obligatory FCoV Eradication Protocol

**Figure 3:**
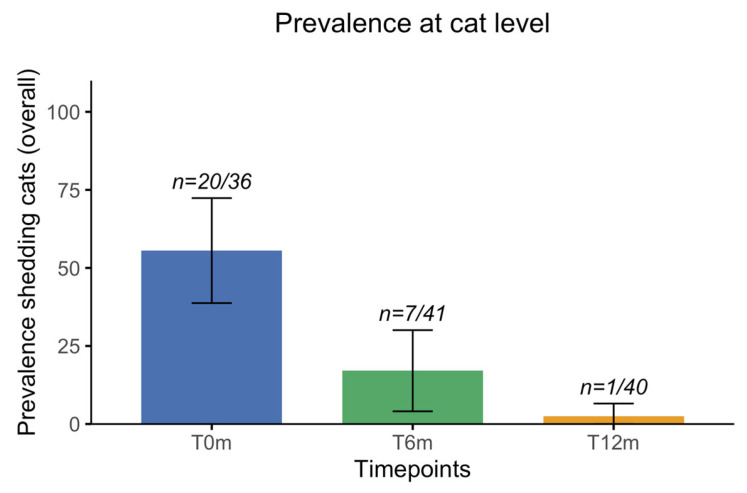
Effect of an obligatory FCoV eradication program on the FCoV shedding in catteries. Bars represent the percentage of cats shedding FCoV at the three sampling timepoints (T0, T6m, T12m). Numbers above the bars represent the ratios of positives to the total tested. Error bars represent the weighted standard deviation (SD), reflecting the variation in prevalence between catteries. The overall effect of time was statistically significant (GLM with binomial distribution and logit link, *p* < 0.001).

As shown in Figure 3, 55.5% of the cats (*n* = 20/36) were shedding FCoV at the start of the study (T0). Following implementation, the proportion of shedders decreased to 17% (*n* = 7/41) at timepoint T6m and 2.5% (*n* = 1/40) at timepoint T12m, with no shedders detected at the final timepoint in all compliant catteries. The overall reduction was statistically significant (GLM, *p* < 0.001).

A linear mixed-effects model confirmed a significant negative effect of time on the outcome (B = −24.65, SE = 4.03, *p* = 0.001) with model assumptions met and no major outliers. The generalized linear model revealed a significant decrease in the probability of shedding (B = −1.877, SE = 0.32, *p* < 0.001), with an odds ratio of 0.153 (95% CI [0.082, 0.287]).

At the cattery level, baseline prevalence was 100% (*n* = 5/5) and dropped to 60% (*n* = 3/5) at T6m and to 20% (*n* = 1/5) at T12m (Table 3). Four out of five catteries achieved their objective to house no shedders and got completely FCoV-free. In fact, after 6 months (T6m), two catteries already achieved this FCoV-free status. Only one cattery was still positive, housing only one shedding cat. This cat was a persistent shedder and was placed into isolation for at least 6 months in order to stop the transmission of the virus to other cats. In contrary to other catteries, this breeder was not motivated to rehome this cat.

The multivariate analysis demonstrated that none of the evaluated risk factors had a significant association with FCoV shedding over time (*p* > 0.05). The number of cats, baseline infection prevalence, viral loads, age, sex, and breed did not significantly influence the successful eradication of the virus under this strict protocol.

## 4. Discussion

This study demonstrates that the implementation of structured management protocols can significantly reduce FCoV shedding in breeding catteries. Several key factors appeared to contribute to this success, including improved hygiene protocols, grouping cats according to their FCoV status, frequent screening, and the use of bentonite as litter material. The identification and isolation, or when necessary, rehoming of persistent shedders turned out to be particularly important.

Almost all participating catteries housed at least one persistent shedder that continued to shed the virus over an extended period. Preventing contact between these animals and the rest of the population effectively interrupted viral circulation and markedly reduced the overall prevalence at the cat level. In several catteries, viral circulation ultimately became restricted to a single persistent shedder, meaning that if this animal was removed or rehomed, the entire cattery immediately became free of FCoV. Unfortunately, not all breeders were willing to take this measure, resulting in six catteries housing only one shedder at T12m.

These observations also provide insight into the shedding dynamics of FCoV within multi-cat environments. The concept of intermittent FCoV shedding has been reconsidered in recent years. While some cats appear to shed FCoV intermittently, this phenomenon is now thought to result from repeated cycles of reinfection rather than true intermittent viral excretion. As suggested by Addie et al. (2023), the apparent fluctuations in shedding status are likely due to the dynamic nature of FCoV transmission within multi-cat settings, where cats are frequently re-exposed to the virus through contaminated litter trays or close contact with infected individuals [28]. Some cats, however, develop persistent infection, continuing to shed virus over extended periods; approximately 13% of naturally infected cats have been identified as persistent shedders [30]. These findings indicate that what has traditionally been interpreted as intermittent shedding may, in fact, represent ongoing reinfection events within a highly endemic environment rather than periodic reactivation of a latent infection.

While viral sequence information was not obtained for every single participating facility, preliminary sequencing data from a subset of the studied catteries confirmed the continuous shedding of the exact same resident FCoV strain over time within those environments, rather than the introduction of new variants. This molecular evidence strongly supports the epidemiologic observation that persistent shedders maintain the infection chain within a cattery. Nevertheless, expanding comprehensive viral sequencing to all facilities in future longitudinal trials would further optimize FCoV prevention and control strategies by mapping precise transmission routes on a wider scale.

The outcomes strongly depended on the level of stringency that was applied. With the voluntary FCoV eradication protocol, the prevalence of shedding cats decreased substantially, yet all catteries remained positive throughout the study. The stimulatory FCoV eradication protocol achieved a more pronounced reduction, with the prevalence falling below 20% of shedders and two catteries successfully clearing FCoV. This suggests that rigorous hygiene combined with targeted isolation strategies can shift the cattery status towards control or near-eradication. Comparable control approaches and results have been described in herd management for other viruses with a similar epidemiological situation like BVDV [31]. Non-cytopathic BVDV is circulating in cattle herds with immunotolerant persistent shedders [32]. This is comparable with FCoV-positive catteries, where persistently shedding cats are taking care of the continuous circulation of FCoV. Once the immunotolerant BVDV-positive animals are identified (RT-qPCR on ear notches) and eliminated from the herd, the circulation of BVDV stops, and the herd becomes negative. This is also the case for FCoV circulation on FCoV-positive catteries. Once the persistent shedders are identified and removed from the cattery, the FCoV circulation stops. The obligatory FCoV eradication protocol was most effective, entirely eliminating shedders within one year. While highly demanding in terms of compliance and logistics, these results show that making catteries FCoV-free is achievable in a very short time. Such outcomes are of major importance, as maintaining a non-shedding population eliminates the risk of FIP emergence within that facility.

Our stratified risk factor analysis demonstrates that the association between baseline variables and FCoV shedding outcomes varies across the three protocol groups. Within the voluntary protocol group, the outcomes were significantly influenced by the initial conditions, as its success was significantly hindered by a high baseline infection prevalence (*p* = 0.016) and specific breed vulnerabilities (*p* < 0.001). Conversely, in both the stimulatory and obligatory groups, none of the evaluated cattery- or cat-level risk factors had a statistically significant influence on the final outcomes (*p* > 0.05).

The motivation of the owners is key to being successful, and the smaller the cattery, the better all measures can be performed. The stringent protocols may be difficult to sustain in larger or commercial catteries, as perseverance is critical to success. Further research is necessary to evaluate long-term sustainability, economic feasibility, and breeder acceptance. It should also be questioned if the ease with which FCoV can be eradicated is strain-dependent, given the high mutation and recombination rates observed among different circulating FCoV isolates. Additionally, pathogenesis work should be performed to understand how FCoV can persist in some cats. Currently, we are investigating the immune evasion of FCoV in cats under endemic conditions.

Maintaining an FCoV-negative status, however, requires sustained vigilance. Several catteries introduced preventive measures aimed at avoiding reintroduction of the virus, such as pre-screening of external stud cats. For instance, one participating cattery tested three potential mating partners before breeding, two of which were identified as FCoV shedders. Temporary isolation after potential exposure events, including breeding, purchase of new animals, or participation in cat shows, was also regarded as an essential biosecurity measure.

While a formal cost–benefit ratio was not mathematically calculated, the economic implications are highly dependent on cattery size. For larger facilities housing around 20 cats or more, repeated RT-qPCR screening represents a substantial financial investment. However, this upfront screening cost must be weighed against the alternative: the devastating emotional and financial impact of a clinical FIP outbreak. Given that modern antiviral therapies for FIP remain exceptionally expensive, investing in strict management and testing protocols to achieve an FCoV-negative status remains a cost-effective long-term strategy to safeguard the breeding program.

Building on these encouraging results, the developed protocol will be implemented more broadly. In collaboration with the Flemish Animal Welfare authorities (Dierenwelzijn Vlaanderen), initiatives are being launched to establish official FCoV status recognition for catteries and to issue certification for kittens at the point of sale. We hope that other countries will launch similar initiatives in order to better control FCoV in the world.

## Figures and Tables

**Table 1:** Number of positive and negative catteries at three sampling timepoints (T0, T6m, T12m). Catteries were considered positive if at least one cat tested FCoV-positive by RT-qPCR.

Timepoint	# of Catteries	# of Positive Catteries ^1^	# of Negative Catteries ^2^
T0	10	10	0
T6m	10	10	0
T12m	10	10	0

^1^ Positive cattery: cattery housing at least 1 shedding cat; ^2^ negative cattery: cattery housing no shedders.

**Table 2:** Number of positive and negative catteries at three sampling timepoints (T0, T6m, T12m). Catteries were considered positive if at least one cat tested FCoV-positive by RT-qPCR.

Timepoint	# of Catteries	# of Positive Catteries ^1^	# of Negative Catteries ^2^
T0	5	5	0
T6m	5	5	0
T12m	5	3	2

^1^ Positive cattery: cattery housing at least 1 shedding cat; ^2^ negative cattery: cattery housing no shedders.

**Table 3:** Number of positive and negative catteries at three sampling timepoints (T0, T6m, T12m). Catteries were considered positive if at least one cat tested FCoV-positive by RT-qPCR.

Timepoint	# of Catteries	# of Positive Catteries ^1^	# of Negative Catteries ^2^
T0	5	5	0
T6m	5	3	2
T12m	5	1	4

^1^ Positive cattery: cattery housing at least 1 shedding cat; ^2^ negative cattery: cattery housing no shedders.

## Data Availability

The data that support the findings of this study are available from the corresponding author upon reasonable request.

## Supplementary Materials

The following supporting information can be downloaded at: https://www.mdpi.com/article/10.3390/v18060614/s1, Supplementary Protocol S1: Voluntary protocol for cat breeders (translated English version); Supplementary Protocol S2: Stimulatory protocol for cat breeders (translated English version); Supplementary Protocol S3: Obligatory protocol for cat breeders (translated English version); Supplementary Figure S1: Accompanying management and virological results for the voluntary protocol; Supplementary Figure S2: Accompanying management and virological results for the stimulatory protocol; Supplementary Figure S3: Accompanying management and virological results for the obligatory protocol.
