# Supplementary material for: Successful Eradication of Feline Coronavirus in Breeding Catteries Paves the Way to Prevent Feline Infectious Peritonitis
Source: Viruses. 2026 May 28;18(6):614. doi: 10.3390/v18060614 (PMC13308486; doi:10.3390/v18060614)
Supplement: Supplementary file 1 [file viruses-18-00614-s001.zip › Supplementary Figure S1.pdf]

Supplementary material Figure S1: Voluntary protocol

General Legend:

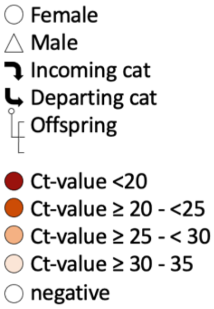

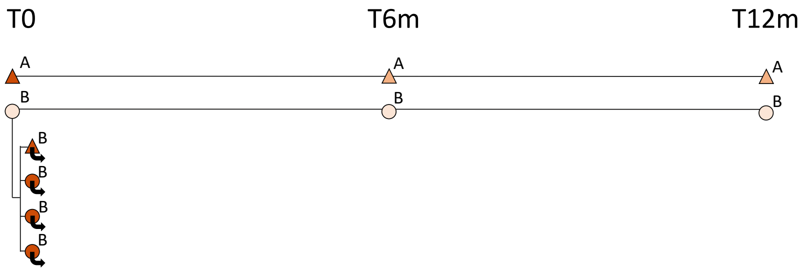

Figure S1.1: Evolution per cat and grouping in cattery B.
A male cat was housed in room A, separately from the female cat (room B). The offspring (T0) was housed together with the queen in room B.

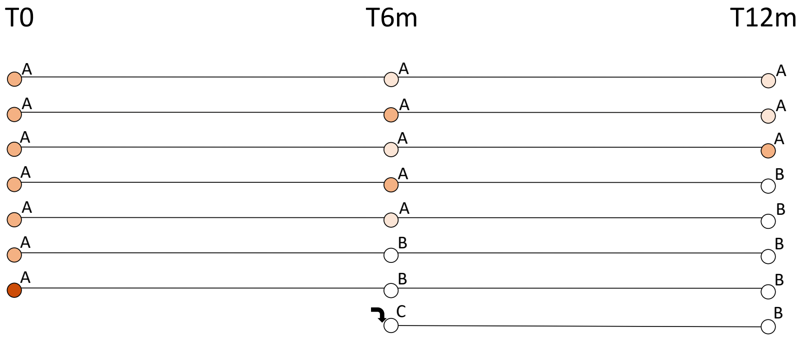

Figure S1.2: Evolution per cat and grouping in cattery C.
At T0, all cats were housed together in room A. At T6m and T12m negative cats were housed in room B. One incoming cat was isolated in room C upon arrival in the cattery. At T12m, this cat was housed together with the other negative cats in room B.

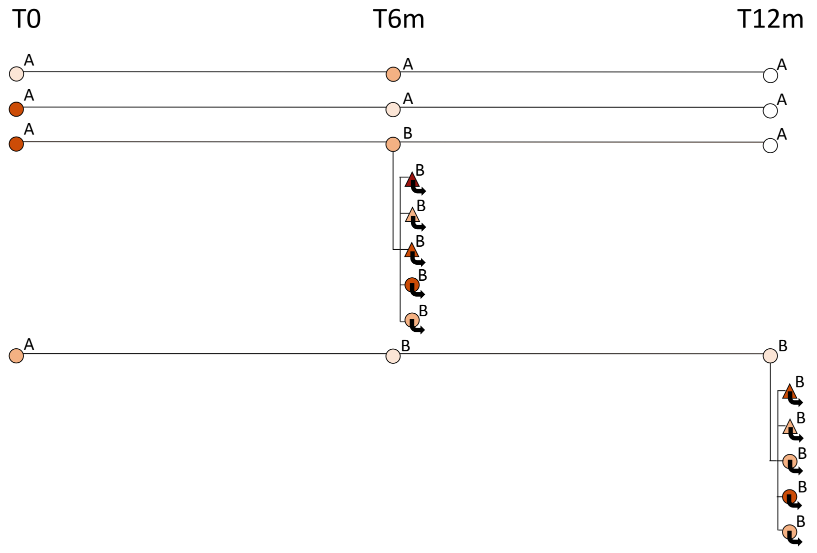

Figure S1.3: Evolution per cat and grouping in cattery E.
All cats were housed together in room A at T0. At T6m, the animals were divided in 2 groups by housing 2 cats and 5 kittens in room B. At T12m, room A only housed negative cats.

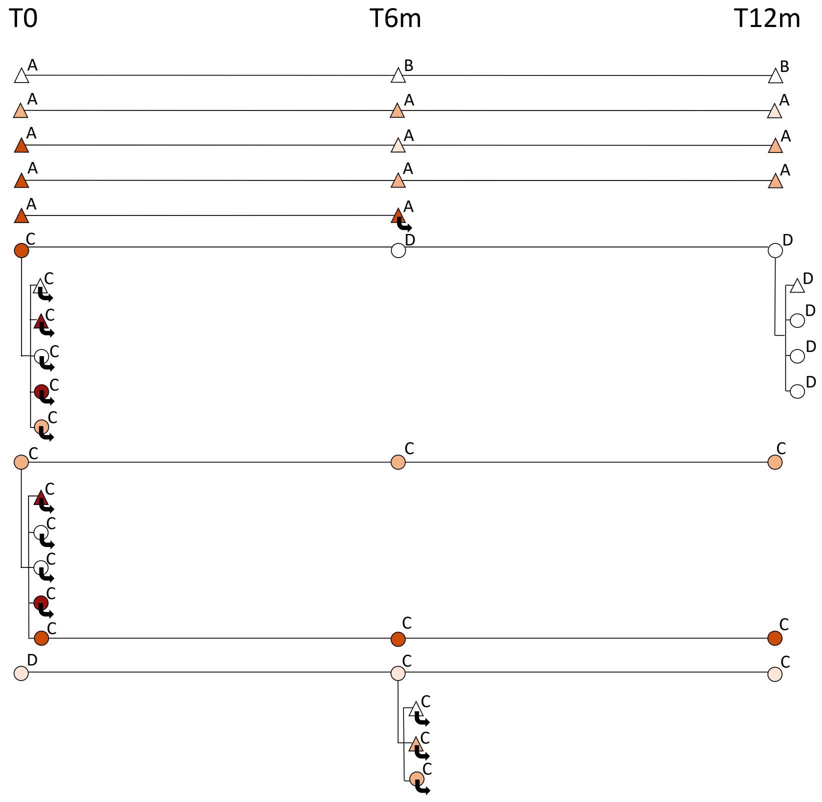

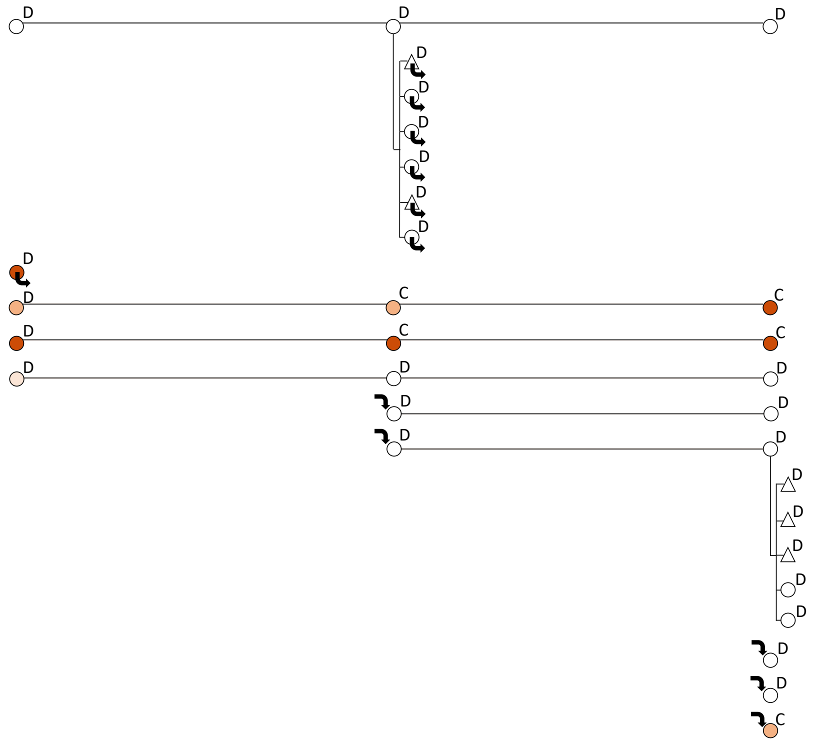

Figure S1.4: Evolution per cat and grouping in cattery J.
At T0, all male cats were housed together in room A and female cats were housed in room C and D. By T6m, one male cat became negative and was housed separately in room B. At T6m, one male cat became FCoV negative and was housed separately in room B. Some female cats also became negative and they were housed in room D; meanwhile positive cats that were housed in room D at T0 were moved to room C at T6m, resulting in a complete FCoV free room (D) at T6m and T12m. Also, room D was used as a queening room for negative mother cats at T6m and T12m, resulting in FCoV free offspring. At T12m, three cats arrived in the cattery: two of them tested negative upon arrival and were housed in room D, one cat tested positive and was housed in room C.

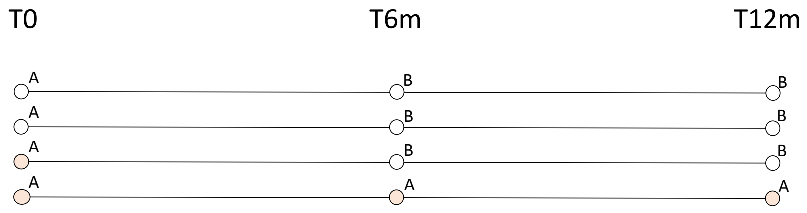

Figure S1.5: Evolution per cat and grouping in cattery K.
At T0, all cats were housed together in room A. At T6m and T12m, positive and negative cats were housed separately in room A and B respectively.

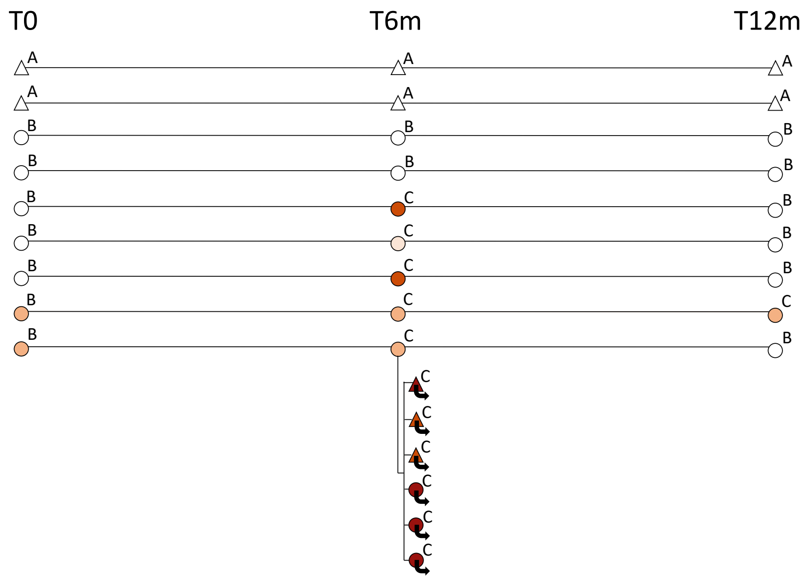

Figure S1.6: Evolution per cat and grouping in cattery L.
At T0, all male cats were housed together in room A; all female cats were housed together in room B. At T6m and T12m, positive cats were separated from negative cats by housing them in room C.

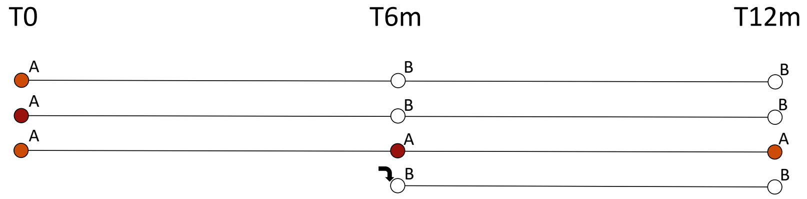

Figure S1.7: Evolution per cat and grouping in cattery N.
At T0, all cats were housed together in room A. At T6m and T12m, positive and negative cats were housed separately in room A and B respectively. One cat arrived in the cattery (T6m) and tested negative, so it was housed in room B.

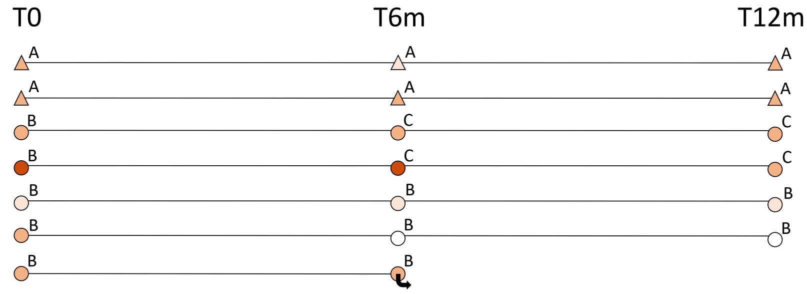

Figure S1.8: Evolution per cat and grouping in cattery P.
At T0, all male cats were housed together in room A, all female cats were housed together in room B. At T6m and T12m, the group of female cats was divided into 2 groups (room B and C).

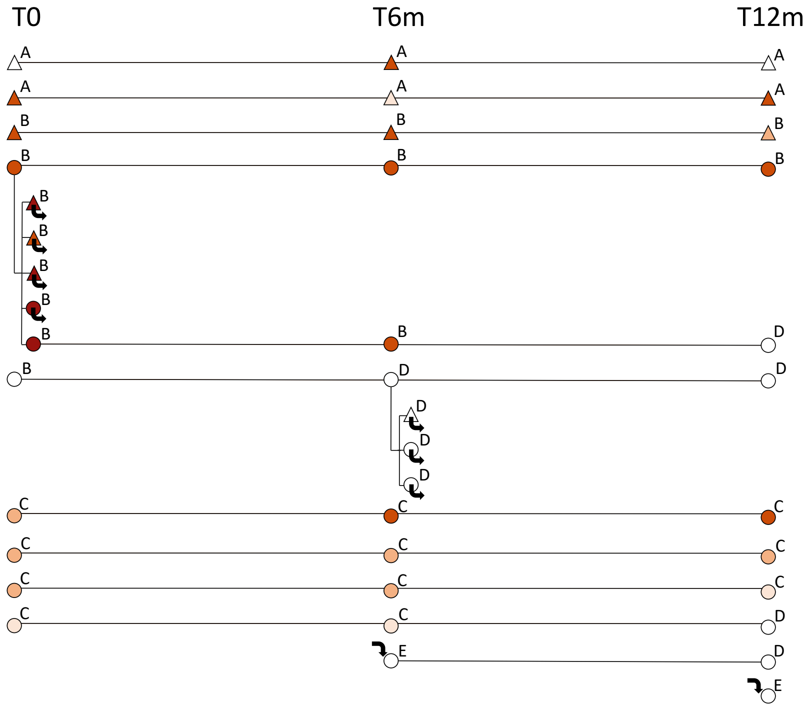

Figure S1.9: Evolution per cat and grouping in cattery S.
Two male cats were housed in room A at the 3 timepoints, one male cat was spayed and was housed with some female cats in room B. Other female cats were housed in room C. At T6m and T12m, negative female cats were housed in room D, separately from positive cats. Incoming cats were first housed in the isolation room (room E) before joining the negative group in room D.

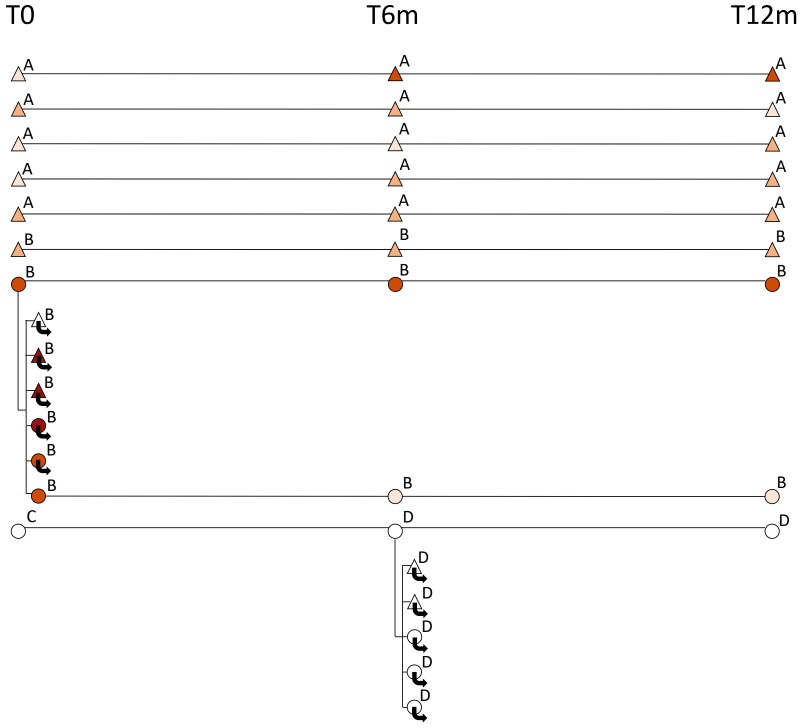

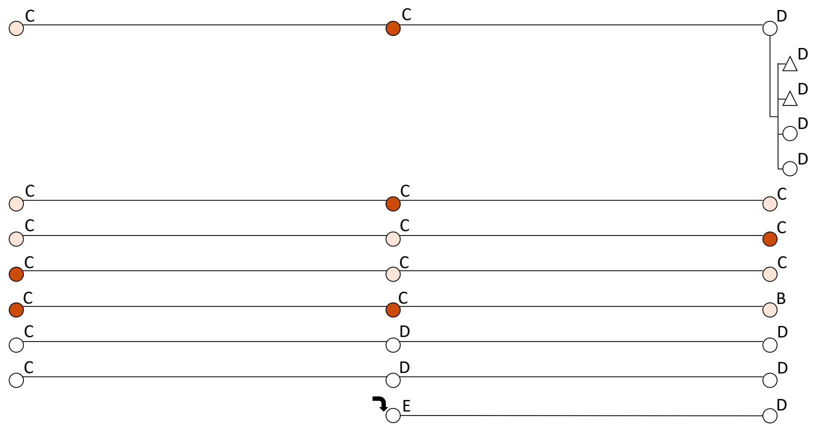

Figure S1.10: Evolution per cat and grouping in cattery T.
Five male cats were housed in room A at the 3 timepoints, 1 male cat was spayed and was housed with some female cats in room B. Other female cats were housed in room C. At T6m and T12m, negative female cats were housed in room D, together with the negative offspring. An incoming cat was first housed in the isolation room (room E) before joining the negative group in room D.
