# Supplementary material for: Successful Eradication of Feline Coronavirus in Breeding Catteries Paves the Way to Prevent Feline Infectious Peritonitis
Source: Viruses. 2026 May 28;18(6):614. doi: 10.3390/v18060614 (PMC13308486; doi:10.3390/v18060614)
Supplement: Supplementary file 1 [file viruses-18-00614-s001.zip › Supplementary Figure S2 .pdf]

Supplementary material Figure S2: Stimulatory protocol

General Legend:

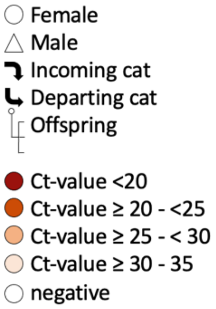

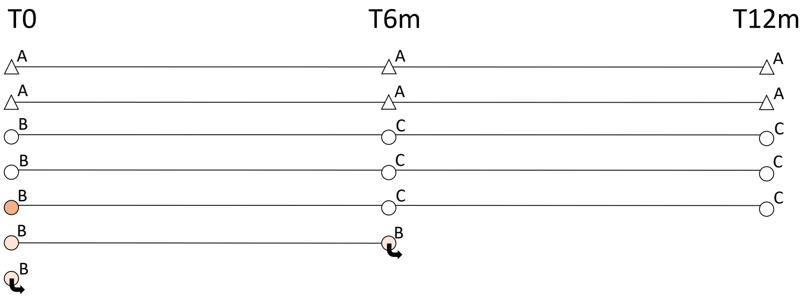

Figure S2.1: Evolution per cat and grouping in cattery F.
At T0, all male cats were housed together in room A separately from the female cats in room B. At T6m, negative female cats were housed in room C and 1 positive cat was housed individually in room B. Unfortunately, this cat stayed positive and left the cattery. Resulting in an FCoV-free cattery at T12m.

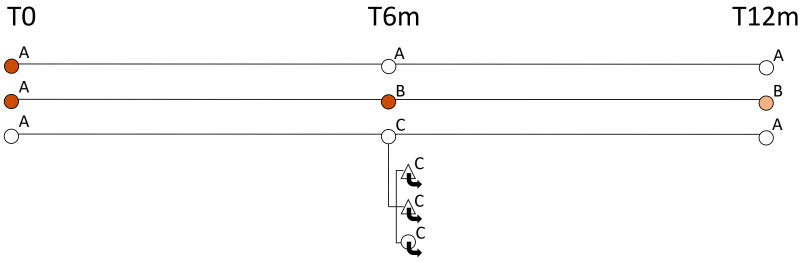

Figure S2.2: Evolution per cat and grouping in cattery G.
At T0m, all cats were housed in room A. At T6m, one positive cat was housed individually in room B separately from the negative cats. Room C was used as a queening room for the negative queen and litter. At T12m, the positive cat was still housed in room B isolated from the negative cats housed in room A.

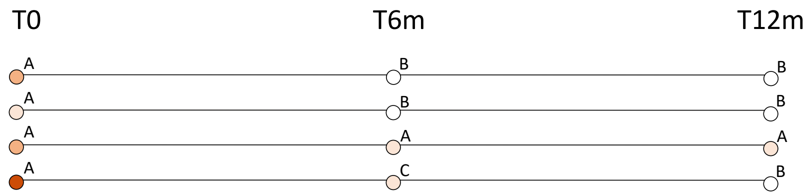

Figure S2.3: Evolution per cat and grouping in cattery M.
All cats were housed together in room A at T0. At T6m, negative cats were housed in room B and the positive cats were housed individually in room A and room C. By T12m, the cat housed in room C became negative and was housed in room B with the other negative cats. The cat housed in room A was still positive and therefore still housed individually in room A.

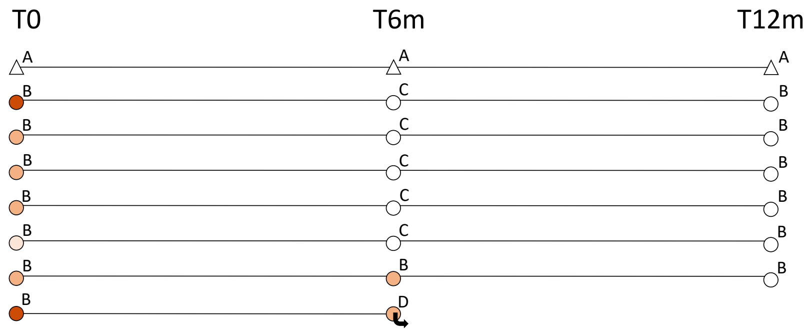

Figure S2.4: Evolution per cat and grouping in cattery O.
At T0, one male cat was housed in room A, separately from all female cats in room B. At T6m, all negative female cats were housed in room C and positive cats were housed individually in room B and D. By T12m, the cat housed in room D stayed positive and left the cattery. The other positive cat (room B) became negative resulting in a FCoV-free cattery at T12m.

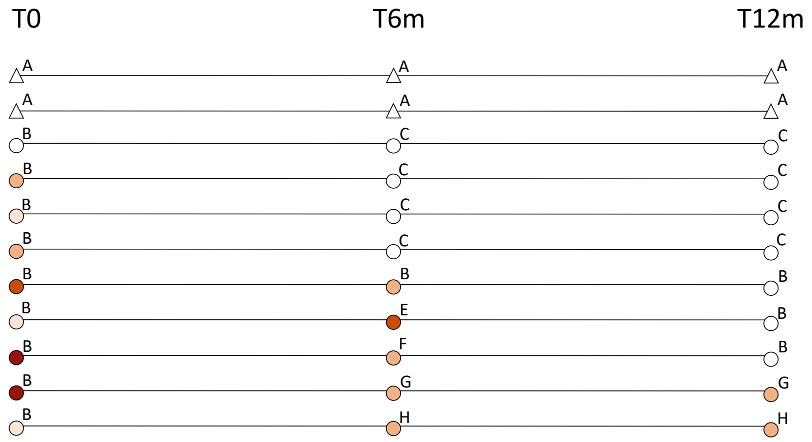

Figure S2.5: Evolution per cat and grouping in cattery R.
At T0, two male cats were housed in room A, separately from all female cats in room B. At T6m, all negative female cats were housed together in room C. The positive female cats were housed individually in room B, E, F, G and H. At T12m, some cats became negative and were housed back in

groups of 4 (room C) and 3 cats (room B). Two cats remained positive and were still housed individually in rooms G and H.
