# Supplementary material for: Successful Eradication of Feline Coronavirus in Breeding Catteries Paves the Way to Prevent Feline Infectious Peritonitis
Source: Viruses. 2026 May 28;18(6):614. doi: 10.3390/v18060614 (PMC13308486; doi:10.3390/v18060614)
Supplement: Supplementary file 1 [file viruses-18-00614-s001.zip › Supplementary Figure S3 .pdf]

Supplementary material Figure S3: Obligatory protocol

General Legend:

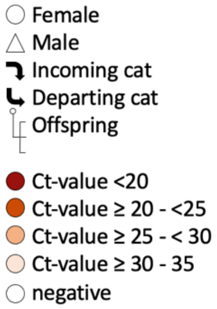

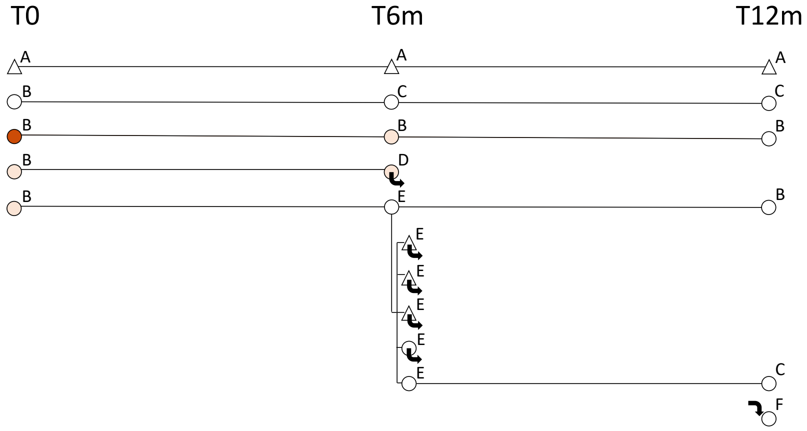

Figure S3.1: Evolution per cat and grouping in cattery A.
At T0, one negative male cat was housed in room A, separately from the female cats in room B. At T6m, all positive cats were housed individually in rooms B and D separately from a single negative cat in room C and a queening cat and her litter in room E (queening room). The positive cat housed in room D left the cattery. At T12m, the positive cat housed in room B became negative and all female cats were grouped in room B and C. One cat arrived in the cattery and was housed in the isolation room (room F), this cat tested negative, resulting in an FCoV-free cattery at T12m.

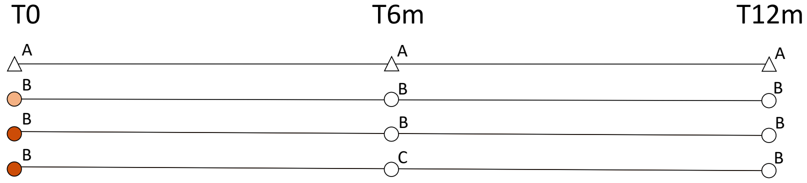

Figure S3.2: Evolution per cat and grouping in cattery D.
At T0, one negative male cat was housed in room A, separately from the female cats in room B. At T6m, one female cat was housed in room C and became negative. The two other cats in room B also became negative resulting in an FCoV-free cattery at T6m. At T12m, the cat housed in room C was housed back in room B with the other cats.

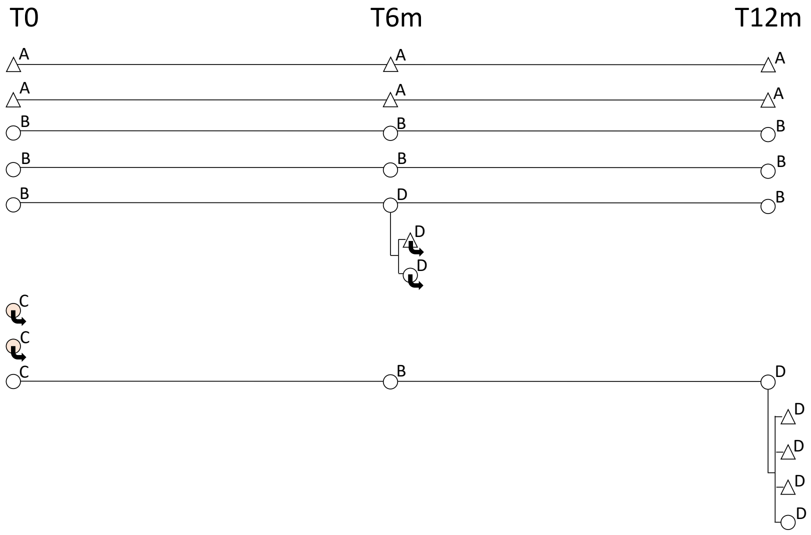

Figure S3.3: Evolution per cat and grouping in cattery H.
At T0m, all male cats were housed together in room A. Female cats were already grouped: three cats in room B and three in room C. Two cats tested positive and immediately left the cattery, resulting in an FCoV-free cattery from T6m. At T6m and T12m, room D was used as a queening room and room B was used to house the other female cats.

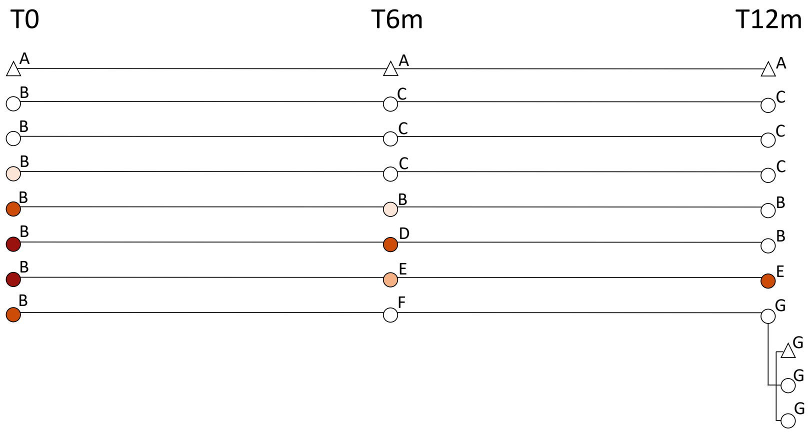

Figure S3.4: Evolution per cat and grouping in cattery I.
At T0, one negative male cat was housed in room A, separately from the female cats in room B. At T6m, all positive cats were housed individually in room B, D and E. Negative cats were housed in room C or F. At T12m, negative cats were housed in room C (2 cats) and room D (2 cats). One cat was still positive and was still housed in room E. Room G was used as a queening room to house a negative queen and her litter.

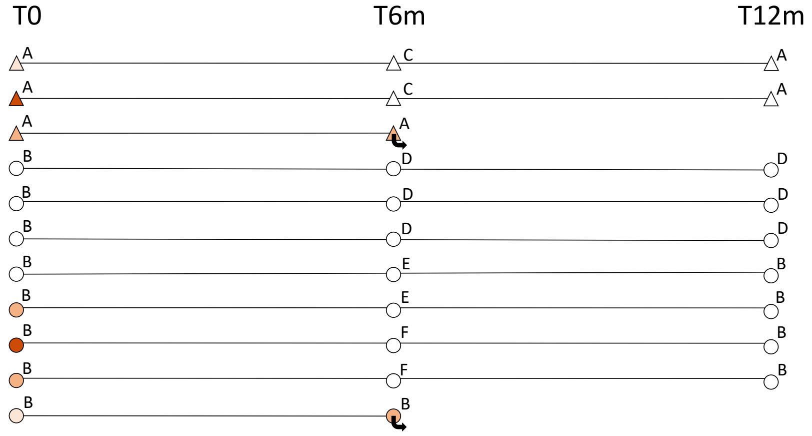

Figure S3.5: Evolution per cat and grouping in cattery Q.
At T0, three male cats were housed in room A, separately from the female cats in room B. At T6m, the negative male cats were housed in room C, separately from the positive male cat in room A. Negative female cats were housed in three groups: three cats in room D, two cats in room E and two cats in room F. One cat remained positive and was still housed individually in room B. By T12m, the positive male and female cats left the cattery resulting in an FCoV-free cattery at this timepoint. The cats were still housed in groups: the male cats in room A and the female cats in room D (3 cats) and B (4 cats).
