# Supplementary material for: Successful Eradication of Feline Coronavirus in Breeding Catteries Paves the Way to Prevent Feline Infectious Peritonitis
Source: Viruses. 2026 May 28;18(6):614. doi: 10.3390/v18060614 (PMC13308486; doi:10.3390/v18060614)
Supplement: Supplementary file 1 [file viruses-18-00614-s001.zip › Supplementary Protocol S1.pdf]

Voluntary FCoV reduction protocol

Guidelines for breeding catteries to reduce FCoV circulation

Table of contents

Introduction **3**

Terms and definitions **4**

General Hygiene Measures *5*

Litter boxes *5*

FCoV screening *5*

Reduction of overpopulation *6*

Incoming cat(s) *7*

Departing cat(s) *8*

Breeding *8*

Outdoor mating *9*

Pregnancy/litter *9*

Shows *10*

Documentation *10*

Introduction

This protocol was drafted following a large-scale study on FCoV management and prevalence in catteries in Flanders. Eighty one percent of the cats were shedding the virus. This extremely high figure poses a serious risk, as 1 to 5% of FCoV-infected cats develop feline infectious peritonitis (FIP), an almost invariably fatal condition. In addition, the study revealed that there is considerable room for improvement in terms of hygiene practices and housing conditions for cats. To reduce the number of FIP cases in Flanders, it was decided to reduce the prevalence of FCoV shedders.

The circulation of FCoV, and by extension the development of FIP, is influenced by several risk factors. The most significant one is the high population density. The risk of transmission increases in environments where multiple cats live together in a limited space. Poor hygiene is another major contributor to viral transmission, as FCoV is primarily spread through fecal-oral contact. As such, both of these risk factors must be controlled as much as possible. The present protocol outlines various biosecurity measures that aimed at reducing population density and improving hygiene. Additionally, it emphasizes the importance of determining the FCoV status of the cattery, which can be monitored via RT-qPCR on rectal swabs.

This document provides a series of recommendations aimed at reducing FCoV shedding in catteries. It is the responsibility of catteries to apply these recommendations as consistently and thoroughly as possible.

Terms and definitions

- FCoV: feline coronavirus
- FIP: feline infectious peritonitis
- FCoV screening: determination of the presence of FCoV in a rectal swab using RT-qPCR.
- RT-qPCR: a diagnostic method that quantifies the number of viral genome copies in a sample.
- Positive cat or shedder: a cat of which the rectal swab is RT-qPCR positive
- Negative cat: a cat of which the rectal swab is RT-qPCR negative
- Persistent shedder: a cat that sheds the virus for months to years and is unable to eliminate the infection.
- Cleaning: the removal of visible dirt from surfaces with a detergent solution.
- Disinfection: the process of inactivating microorganisms.
- Sanitary entry zone: a demarcated zone to prevent contamination or infection between two areas.
- Separation: strictly housing cats separately according to their FCoV status, or if their status is unknown.
- Isolation: upon arrival at a facility, cats are strictly isolated from other cats in a dedicated room (quarantine). They are tested 10 days after arrival and must stay there until the test results are known.

General Hygiene Measures

If no measures are taken, FCoV can persist in the environment for up to 7 weeks. Therefore, proper hygienic management of the environment is essential in combatting the virus. Catteries are advised to:

- Equip floors and surfaces with materials that are easy to clean. For example, smooth flooring is preferred, as wood and carpets are more difficult to clean and therefore less suitable.
- Clean floors and surfaces of materials daily. This includes removing visible dirt and contaminants, typically by cleaning with a soap solution.
- Disinfect floors and surfaces of materials at least once a week. This disinfection step should always be preceded by cleaning to optimize the efficacy of the disinfectant.
- Work from clean to dirty areas. For instance, clean and disinfect food and water bowls first, and only then proceed to the litter boxes.
- Work from FCoV-negative to FCoV-positive status. Start by cleaning areas housing FCoV-negative animals before moving on to those with positive animals.
- Install a sanitary entry zone in every room where cats are housed. Ideally, this includes:
    - Washing hands when entering and leaving the room.
    - Wearing gloves while inside the room.
    - Changing footwear before entering the room.
      Alternative: wearing overshoes.
      Alternative: installing disinfectant baths at the entrance of the room.
    - Not sharing equipment or materials between different rooms.

Litter boxes

Litter boxes are the primary source of infection, as the virus is transmitted via the fecal-oral route. Therefore, maintaining proper litter box hygiene is crucial. Catteries are advised to:

- Provide at least one litter box per cat, plus one extra.
- Assign a separate scoop to each litter box.
- Use litter with bentonite as the main component, as this substance has virus-inactivating properties.
- Remove feces from the litter box daily.
- Clean and disinfect litter boxes and scoops at least once a week.
- Use gloves and wash hands after every contact with the litter box.
- Place the litter box as far away as possible from food and water bowls, ideally in a separate room, to help prevent fecal-oral transmission.

FCoV screening

It is essential to know the FCoV status of the cats in the facility. This allows shedders to be kept separate, reducing viral transmission to other cats. Detection of FCoV is done via RT-qPCR on rectal swabs. Catteries are advised to follow the flowchart below:

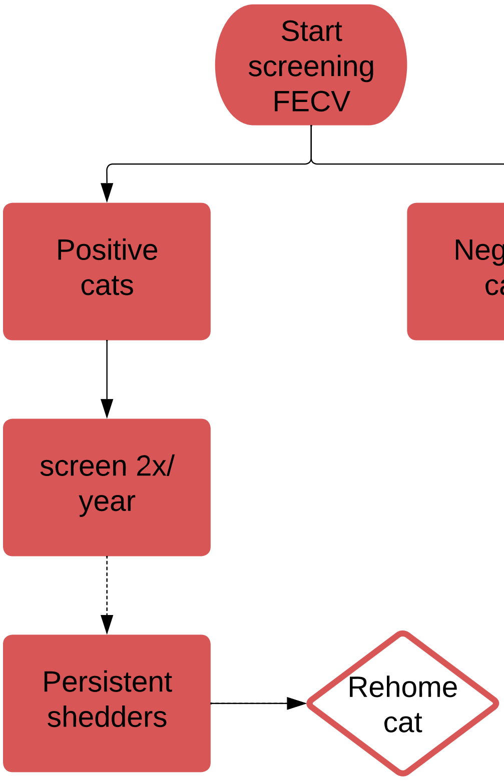

Flowchart 1: General FCoV screening strategy on a cattery

Ideally, all cats in the cattery should be tested when FCoV screening is initiated. Cats that test positive should, where possible, be isolated from negative animals. Preferably, they are housed individually; if that is not feasible, they may be placed in a positive group of up to five cats. These virus shedders should ideally be screened individually at least twice a year. If they test negative, they may join a group of FCoV-negative animals.
To identify persistent shedders, positive cats must be housed individually for at least 4 months. If they continue to shed the virus for at least 4 months, this is strongly indicative of a persistent infection. In such cases, it is recommended that these cats be rehomed to individual households (provided they are sterilized/neutered).
Cats that test negative do not need to be retested. These cats may be housed in negative groups of up to five cats.

Reduction of overpopulation

Because catteries often house too many cats at once, there is a significant risk of FCoV circulation. Therefore, overcrowding must be reduced. Catteries are advised to:

- House less than 10 cats, including kittens.
- Isolate cats based on their FCoV status, in groups of no more than 5 cats. An exception is made for kittens, with or without their mother (see chapter on pregnancy/litters).
- House FCoV-negative cats together with other negative cats, separated from positive cats.

- Place FCoV-positive cats in individual isolation whenever possible. If this is not feasible, house them in positive groups, with a maximum of five cats per group.
- Rehome persistent shedders to individual households.
- Maintain documentation listing all grouped animals (see chapter on documentation).

Incoming cat(s)

Introducing new cats into the facility increases the risk of bringing in an infected animal. For this reason, the following guidelines are recommended to reduce the introduction of FCoV. Additionally, the cattery is advised to follow the flowchart below during the introduction of a new cat:

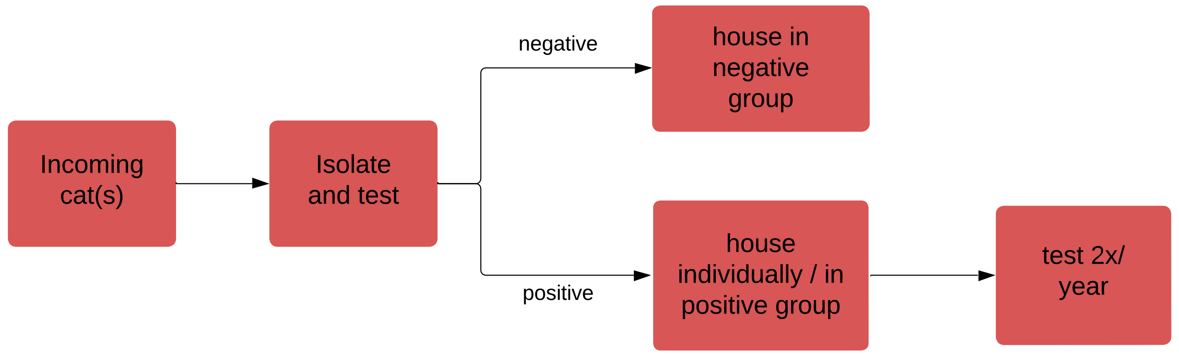

Flowchart 2: FCoV management of incoming cat(s)

New cats should ideally enter the facility in strict isolation. In addition, it is recommended that each cat is tested 10 days after arrival in this space and stays there until the test result is known.

After the isolation period:

- Cats that test negative may join a group of negative animals (maximum of 5 cats). These cats do not require further screening.
- Cats that test positive may, after the isolation period, be housed individually or join a positive group (maximum of 5 cats). Positive cats should be screened twice a year (see FCoV screening).

The isolation room should ideally be:
- Separated from other rooms with animals and the public and located away from busy areas.
- Well ventilated in such a way that the spread of pathogens to other areas of the facility is prevented.
- Equipped with a sanitary entry zone. Ideally, this includes:
    - Washing hands when entering and leaving the room.
    - Wearing gloves while inside the room.
    - Changing footwear before entering the room.
      Alternative: wearing disposable shoe covers.
      Alternative: using disinfectant footbaths at the entrance.

  - No sharing of equipment between different rooms.
- Accompanied by documentation listing all hygiene measures taken in the room (see chapter on documentation).

After the animals leave the isolation room, the space should ideally be cleaned and disinfected thoroughly. Additionally, it is recommended to keep the room vacant for at least one week before placing new cats into isolation there.

Departing cat(s)

When an individual cat leaves a group of either FCoV-negative or FCoV-positive animals, no additional hygiene measures are required.
However, when a positive cat that was housed individually, or a litter or group of cats that were isolated together, leaves the space, the cattery is advised to:

- Thoroughly clean and disinfect the area where the cats were housed.
- Keep the room vacant for at least one week after the cats departed.

This reduces the risk of the virus persisting in the environment and potentially infecting other cats.

Breeding

Since there is a genetic component involved in the mutation from FCoV to FIP-associated FCoV, the selection of breeding cats is very important. The cattery is advised to follow the following flowchart:

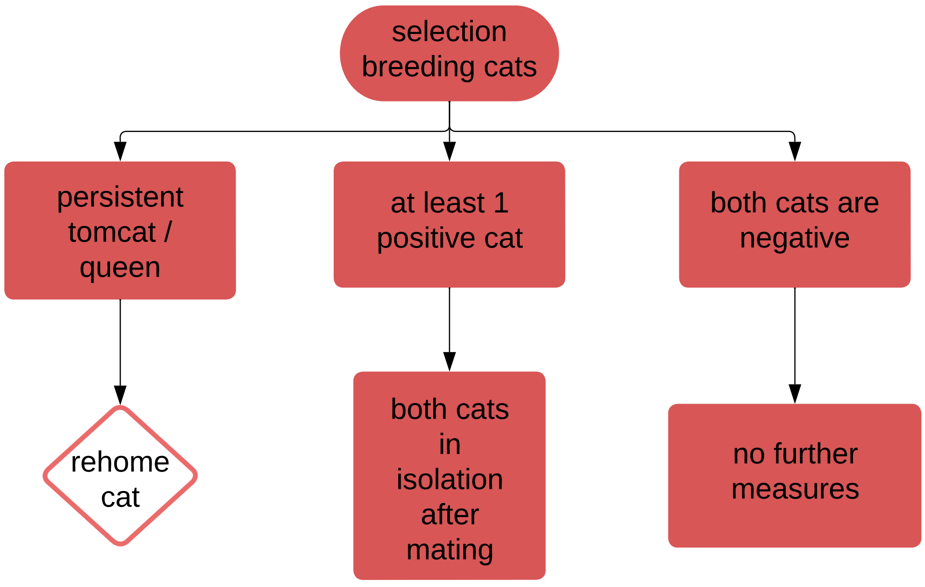

Flowchart 3: FCoV management of breeding cats

It is advised not to breed with persistent shedders, as they pose a risk for continuous (re)infection of other cats. Ideally, these cats should be rehomed to individual households. If at least one of the mating partners is positive, it is recommended to isolate both animals after mating. Preferably, these cats should be tested after 10 days of isolation. Ideally, breeding should be done with two negative cats. This way, there is no risk of virus transmission during the mating period.

Additionally, it is advised to:

- Exclude males or females that have previously produced one or more litters with FIP kittens from breeding.
- Avoid outdoor mating, as this poses a risk of introducing FCoV into the cattery.
- Document the mating (see documentation chapter).

Outdoor mating

When mating occurs outdoors, there is a risk that an external strain of FCoV may enter the cattery. To minimize this risk, the cattery is advised to:

- Breed with cats that are both FCoV-negative.
- Provide at least two litter boxes on site. These should preferably be placed as far apart as possible and away from food and water bowls. The use of shared litter boxes is strongly discouraged.
- Isolate both cats after mating and test them for FCoV. Both animals should be treated as "incoming cats" to the cattery, so ideally flowchart 2 is followed.
- Document the mating (see documentation chapter).

Pregnancy/litter

Young cats are particularly susceptible to developing FIP. Therefore, it is very important to delay FCoV infection, and consequently the chance of mutation to FIP-associated FCoV, for as long as possible. To protect kittens from FCoV infection, the following guidelines should be followed:

- Isolate the pregnant queen from FCoV-positive cats.
- Provide a sanitary entry zone in the area where the mother and her litter stay. This entry zone includes:
    - Washing hands upon entering and leaving the room.
    - Wearing gloves inside the room.
    - Changing footwear when entering the room.
      Alternative: wearing disposable shoe covers.
      Alternative: placing disinfectant footbaths at the entrance.
    - Not sharing equipment or materials between different rooms

Shows

Since cats from different households come together here, there is a risk of infection. Therefore, the cattery is advised to:

- Avoid attending shows with (persistent) shedders.
- Do not use shared litter boxes or equipment on site.
- Isolate the cat upon return and test for FCoV. The cat should be treated as an “incoming cat” to the cattery, so flowchart 2 should be followed accordingly.

Documentation

Catteries are also advised to keep certain documents, either electronically or on paper. This documentation supports the structural aspects of FCoV management. Catteries are recommended to create and maintain the following records:

- A logbook of all cats housed in the cattery and their test results.
- A record for each cat in isolation.
- A record of all (outdoor) matings involving at least one parent from the cattery.
