# Supplementary material for: Successful Eradication of Feline Coronavirus in Breeding Catteries Paves the Way to Prevent Feline Infectious Peritonitis
Source: Viruses. 2026 May 28;18(6):614. doi: 10.3390/v18060614 (PMC13308486; doi:10.3390/v18060614)
Supplement: Supplementary file 1 [file viruses-18-00614-s001.zip › Supplementary Protocol S3.pdf]

# Obligatory FCoV eradication protocol

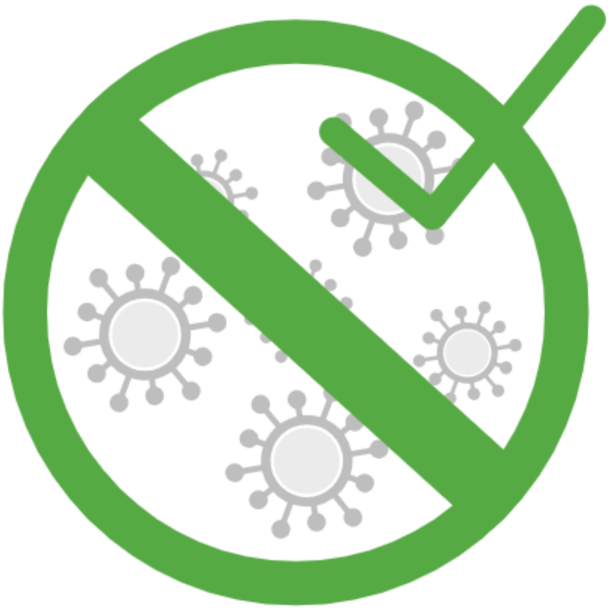

## Table of contents

*Introduction* ........ 3
*Terms and definitions* ........ 4
*Infrastructure* ........ 5
*General Hygiene Measures* ........ 5
*Litter Boxes* ........ 6
*FCoV screening* ........ 6
*Incoming cat(s)* ........ 7
*Departing cats* ........ 8
*Breeding* ........ 8
*Outdoor mating* ........ 8
*Pregnancy / Litter* ........ 8
*Shows* ........ 9
*Documentation* ........ 9

## Introduction

This protocol was drafted following a large-scale study on FCoV management and prevalence in catteries in Flanders. Eighty one percent of the cats were shedding the virus. This extremely high figure poses a serious risk, as 1 to 5% of FCoV-infected cats develop feline infectious peritonitis (FIP), an almost invariably fatal condition. In addition, the study revealed that there is considerable room for improvement in terms of hygiene practices and housing conditions for cats. To reduce the number of FIP cases in Flanders, it was decided to reduce the prevalence of FCoV shedders.

The circulation of FCoV, and by extension, the development of FIP, is influenced by several risk factors. The most significant one is the high population density. The risk of transmission increases in environments where multiple cats live together in a limited space. Poor hygiene is another major contributor to viral transmission, as FCoV is primarily spread through fecal-oral contact. As such, both of these risk factors must be controlled as much as possible. The present protocol outlines various biosecurity measures that aimed at reducing population density and improving hygiene. Additionally, it emphasizes the importance of determining the FCoV status of the cattery, which can be monitored via RT-qPCR on rectal swabs.

This document provides a series of strict measures that is necessary to stop the circulation of FCoV in catteries.

## Terms and definitions

- FCoV: feline coronavirus
- FIP: feline infectious peritonitis
- FCoV screening: determination of the presence of FCoV in a rectal swab using RT-qPCR.
- RT-qPCR: a diagnostic method that quantifies the number of viral genome copies in a sample.
- Positive cat or shedder: a cat of which the rectal swab is RT-qPCR positive
- Negative cat: a cat of which the rectal swab is RT-qPCR negative
- Persistent shedder: a cat that sheds the virus for months to years and is unable to eliminate the infection.
- Cleaning: the removal of visible dirt from surfaces with a detergent solution.
- Disinfection: the process of inactivating microorganisms.
- Sanitary entry zone: a demarcated zone to prevent contamination or infection between two areas.
- Separation: strictly housing cats separately according to their FCoV status, or if their status is unknown.
- Isolation: upon arrival at a facility, cats are strictly isolated from other cats in a dedicated room (quarantine). They are tested 10 days after arrival and must stay there until the test results are known.

## Infrastructure

To prevent FCoV circulation within the facility, the cattery must be equipped with the following rooms:

- Multiple rooms for housing cats. The cattery must have enough rooms to individually house all FCoV-positive cats and to house all negative cats in groups of no more than 3 animals.
- At least 1 isolation room: This room is exclusively intended for housing cats that are entering the cattery and cats that may have been in contact with an FCoV-positive cat (e.g., after shows or (outdoor) mating). Cats are tested 10 days after arrival in this room and must stay there until the test result is known. Cats housed here are not considered part of the cattery and may not be used for breeding. This isolation room must be:
    - Separated from other rooms with animals and the public and located away from busy areas.
    - Well-ventilated in a way that prevents transmission of pathogens to other areas of the facility.
    - Equipped with documentation for each cat in isolation, including the reason for isolation (see Documentation section).
- At least 1 queening room: a separate room exclusively intended for housing a (pregnant) queen and her kittens.

These rooms must be strictly separated from each other and all must be equipped with a sanitary entry zone. In this entry zone one has to:

- Wash hands when entering and leaving the room.
- Wear gloves inside the room.
- Change footwear upon entering the room.
    - Alternative: wearing overshoes.
    - Alternative: installing disinfectant baths at the entrance of the room.

No materials may be shared between different rooms.

## General Hygiene Measures

FCoV can persist in the environment for up to 7 weeks if no measures are taken. Proper environmental hygiene is therefore critical in controlling the virus. The cattery must meet the following criteria:

- Floors and material surfaces have to be easy to clean.
- Floors and material surfaces should be cleaned daily, meaning the removal of visible dirt and contamination.
- Floors and material surfaces have to be disinfected at least twice a week. This disinfection step should always follow cleaning to optimize the effectiveness of the disinfectant.
- Cleaning should be performed from clean to dirty: first the food and water bowls, then the litter boxes.

- Cleaning should be performed from low-risk to high-risk areas: first negative animals, then the isolation room, and finally positive animals.

## Litter Boxes

Litter boxes are the primary source of infection since the virus is transmitted via the orofaecal route. Achieving proper litter box hygiene is therefore crucial. The cattery has to meet the following criteria:

- The number of litter boxes has to be at least equal the number of cats + 1.
- Each litter box needs to have its own scoop.
- Faeces must be removed from litter boxes daily.
- Litter must be primarily composed of bentonite.
- The entire contents of the litter boxes have to be replaced weekly.
- Litter boxes and scoops have to be cleaned and disinfected at least twice a week.
- Hands must be washed after contact with litter boxes.

## FCoV screening

It is essential to know the FCoV status of the cats in the facility. This allows shedders to be kept separate, reducing viral transmission to other cats. Detection of FCoV is done via RT-qPCR on rectal swabs. The cattery is required to follow the flowchart below:

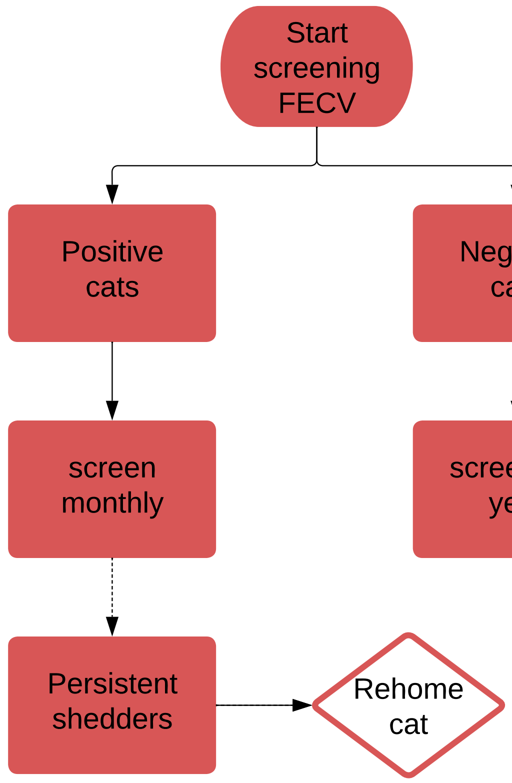

*Flowchart 1: General FCoV screening strategy on a cattery*

Every cat in the cattery must be screened.
**Negative cats** are subsequently tested twice a year. These cats are housed individually or in groups of no more than 3 animals. An exception applies to kittens, with or without the mother (see Pregnancy/Litter section).
**Positive cats** are tested subsequently every month and must be housed individually. If a test comes back negative, the cat may join a group of negative cats.
When cats remain positive while being isolated for at least 4 months they are considered as **persistent shedders** and pose a risk of continuous (re)infection to other cats. These cats must be relocated to an individual household (after spaying/neutering) or another cattery.

Additional requirements:

- Strict isolation has to be maintained between groups. This means housing groups in separate rooms and reducing the risk of cross-contamination through a sanitary entry zone.
- Documentation has to be maintained listing all cats present in the cattery (see Documentation section).

## Incoming cat(s)

Introducing new cats into the cattery increases the risk of bringing in an infected cat. For this reason, the following measures should be followed to reduce the introduction of FCoV. The cattery is required to follow the flowchart below during the introduction of a new cat:

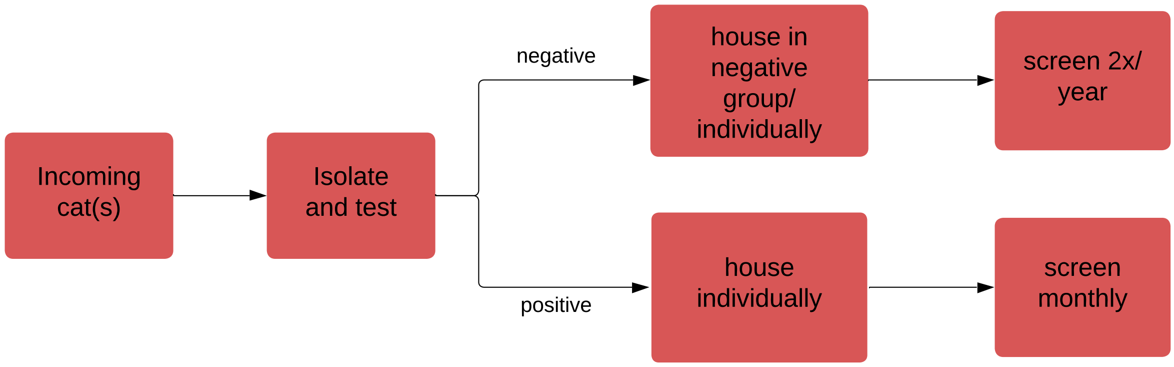


*Flowchart 2: FCoV management of incoming cat(s)*

New cats enter the facility into isolation. Additionally, every cat is tested after 10 days upon arrival at the cattery. After the isolation period:

- Cats that test negative must join a group of negative cats (maximum 3 animals) or, if possible, be housed individually. These cats are screened twice a year.
- Cats that test positive are placed individually after the isolation period and are screened monthly.

* Isolation means strictly separating the cat(s) from other cats in a dedicated room specifically for this purpose (see Infrastructure section). Cats are tested 10 days after arrival in the isolation room and must stay there until the test results are known. Cats in isolation are not considered part of the cattery, may not be used for breeding, and may not attend shows.

After the cats leave the isolation room, the room has to be cleaned and disinfected, and has to remain vacant for at least 2 weeks before other cats are placed in isolation there.

## Departing cats

When any cat or group of separately housed cats leaves the cattery, the facility is responsible for the following measures:

- Thorough cleaning and disinfection of the room(s) where the cats were housed.
- Minimum 2 weeks vacancy of the room(s) after the cats departed.

## Breeding

Since there is a genetic component involved in the mutation of FCoV to FIP-associated FCoV, the selection of parent cats is extremely important. The following measures must be observed. The cattery is also required to follow the measures below:

- Mating only occurs with 2 negative cats.
- Male or female cats that have produced at least one offspring that developed FIP are excluded from breeding.
- After mating, both cats must be placed in isolation and tested for FCoV. Both cats are treated as incoming cats, so Flowchart 2 must be followed.
- All matings must be documented (see Documentation section).

## Outdoor mating

Outdoor matings pose a risk of introducing an FCoV strain from outside the cattery. The following criteria must therefore be observed:

- Do not perform outdoor mating with a positive cat or a cat still in isolation.
- Provide at least 2 litter boxes on-site, placed as far away as possible from food and water bowls.
- After mating, both cats must be placed in isolation and tested for FCoV. Both cats are treated as incoming cats, so Flowchart 2 must be followed.
- Outdoor matings must be documented (see Documentation section).

## Pregnancy / Litter

Young cats are particularly susceptible to develop FIP. It is therefore critical to minimize FCoV infection and, consequently, the risk of mutation to FIP-associated FCoV. To protect kittens from FCoV exposure within the cattery, the facility has to comply with the following criteria:

- The pregnant queen is placed in the queening room 2 weeks before giving birth.
- Only the kittens and their mother may be housed in the queening room. The number of cats may exceed 3 in this room.
- A sanitary entry zone should be provided in the queening room.
- All kittens should be tested for FCoV shedding at the age of 12 weeks.
- After all animals left the queening room, the room has to be cleaned and disinfected, and remain vacant for at least 2 weeks before housing other cats.

## Shows

As cats from different households come together at shows, there is a risk of infection. The cattery must therefore comply with the following criteria:

- Do not attend shows with positive cats or cats in isolation.
- Upon return, the cat must be placed in isolation and tested for FCoV. The cat is treated as an incoming cat, so Flowchart 2 has to be followed.

## Documentation

Catteries must maintain several records, either electronically or on paper. Proper documentation supports the structural management of FCoV. The cattery is required to have the following documents and update them with each change:

- A logbook of all cats housed in the cattery and their test results.
- A record for each cat in isolation.
- A record of all (outdoor) matings involving at least one parent from the cattery.
